# Effect of Basalt Fiber Content and Length on the Strength and Crack Development of Polyvinyl Alcohol/Basalt Hybrid Fiber-Reinforced Cement Soil

**DOI:** 10.3390/polym15092146

**Published:** 2023-04-30

**Authors:** Yonghua Shu, Jingshuang Zhang

**Affiliations:** 1 School of Civil Engineering and Architecture, Anhui University of Science and Technology, Huainan 232001, China; 2021200385@aust.edu.cn; 2 Engineering Research Center of Underground Mine Construction, Ministry of Education, Huainan 232001, China

**Keywords:** cement soil, unconfined compressive strength, basalt fiber, polyvinyl alcohol (PVA) fiber, DIC

## Abstract

Polyvinyl alcohol (PVA) fiber is widely used in geotechnical engineering because of its excellent physical and mechanical properties; however, PVA fibers are prone to aging, while basalt fiber has a natural anti-aging ability, which can be added to cement material to effectively eliminate the effects of aging on PVA fiber. Previous experiments identified that the optimum content of PVA fiber is 0.5% (mass fraction, the same below). Based on this, we continued to add basalt fibers of different lengths (3 mm, 6 mm, 9 mm, 12 mm, 18 mm, 30 mm) and different contents (0%, 0.25%, 0.5%, 0.75%, 1%) to study the effect of both length and content of basalt fibers on the strength of cement soil specimens. It was concluded that adding 0.5 % of 9 mm basalt fiber results in the greatest increase in unconfined compressive strength (UCS). The UCS reached 12.59 MPa, which was 71% higher than specimens without fiber, and a regression analysis was carried out to obtain the relationship among them. The ratio of cement soil in the highest UCS and the relationship among the UCS, the length, and the content of basalt fiber can be used as a reference for practical projects. In addition, digital image correlation (DIC) technology was used to analyze the surface cracks and horizontal strain field when the peak strain was reached at each content and length of the basalt fiber. Finally, the curing mechanism for hybrid fiber cement soil was analyzed by combining the results of the UCS test, DIC test, and SEM test.

## 1. Introduction

In recent years, polyvinyl alcohol fiber (PVA) has attracted a great deal of attention due to its excellent physical and mechanical properties in various cementitious composites [1,2,3,4,5,6,7,8,9,10]. The addition of PVA fiber can increase the compressive strength, tensile strength, and flexural strength of cementitious composites [11,12,13,14,15,16]. However, there is a risk of aging under the effects of harsh environmental factors, which may lead to the failure of infrastructure. As a non-biologically active material, basalt fiber has a natural anti-aging ability [17,18,19,20]. Adding basalt fiber to cement soil material can effectively eliminate the influence of PVA fiber aging, thereby improving the stability of cement–soil materials. This method deserves more application and attention in geotechnical engineering.

In the eastern coastal areas of China, soft clay is widely distributed. Nowadays, with the expansion of cities, a large amount of infrastructure needs to be built in these areas, so methods for improving the mechanical properties of these soil are worthy of our study. In recent years, scholars have added different fibers to cement soil to improve the performance of cement soil. A large number of studies have shown that adding PP, PE, and PVA fibers to cement soil can improve the compressive and tensile properties of cement soil [1,16,21,22,23,24,25,26]. Tang et al. [27] found that PP fiber can improve the peak strength of soil in tensile tests and reduce soil cracking significantly. Yuan et al. [28] used glass fiber and liquid-modified polymer (SH polymer) to reinforce granite residual soil and found that when the dosing of SH polymer was 3.5%, there was a tighter bond between the granite residual soil particles and flaky clay particles, and the impact resistance of the specimens reached a maximum value of 32.16 KN when the content of glass fiber was 3.0%. Govindarajan et al. [29] found that silica nanoparticles and banana fibers can work together to enhance the mechanical properties of soils, with silica nanoparticles increasing the strength of soils with banana fibers to some extent, and banana fibers avoiding brittle damage in soils with silica nanoparticles. Zhao et al. [30] added four kinds of steel fiber content (0%, 1%, 2%, 3%) to concrete and then carried out SHPB tests under four high strain rates (40–220 s^−1^). The test results showed that with the increase in the content of steel fibers, the degree of damage to the specimens showed a significant decline in the trend; the dynamic compressive strength of concrete with the strain rate is the most sensitive, and the dynamic compressive strength has a large change when the strain rate changes slightly; and when the content fiber of 1%, the dynamic compressive strength reached the maximum. Khan et al. [31] added basalt fibers with different lengths and contents to concrete, and the best mechanical properties of concrete were found with the addition of basalt fibers with a length of 12 mm and a content of 0.45%. In addition, the digital image correlation (DIC) technique was first proposed by Gibson and first applied in practice by Kim and Wen as a simple and efficient method of measuring fracture energy. DIC technology is also increasingly used in the strength test for cementitious composites, which can simply and effectively monitor the displacement field and strain field of materials under loads [32]. Zhou et al. [33] examined the whole process of the UCS test for granite with pre-existing defects using DIC technology and AE. The complete cracking process of granite was divided into six grades and distinguished by five characteristic stresses. Xu et al. [34] collected the strain field of a cement soil specimen with jute and PVA fibers in a dry environment using the DIC method and analyzed the evolution of cracks in the cement soil under this condition. Kouta et al. [35] used the DIC technique to obtain the strain and displacement fields of concrete specimens with and without the addition of flax fibers, and it was found that the addition of fibers contributed more to the early cracking in the concrete specimens and large cracks were not found in the concrete specimens with the addition of flax fibers.

The ultimate goal of this study is to propose a high-strength hybrid fiber cement soil. Thus, we innovatively add different lengths and different amounts of basalt fiber on the same basis as adding PVA fiber. The addition of basalt fiber can effectively solve the problem of PVA fiber failure in harsh environments, and the advantages of the two kinds of fibers can be used at the same time to carry out UCS tests on a large number of cement soil specimens with different dosages and obtain the maximum strength. Then, the dosage corresponding to the maximum strength can be applied in engineering. In addition, this study innovatively applies DIC technology to experiments using the cement soil material performance test and analyzes the cracks created in the process of compression failure with the help of DIC technology. Previously, few scholars used DIC technology in experiments on cement soil material. Thus, this study is very meaningful. Finally, an SEM test is carried out to analyze the solidification mechanism of cement soil from a microscopic point of view. The test results can provide some reference for the improvement of soft foundations.

## 2. Experiment

### 2.1. Materials

**Figure 1:**
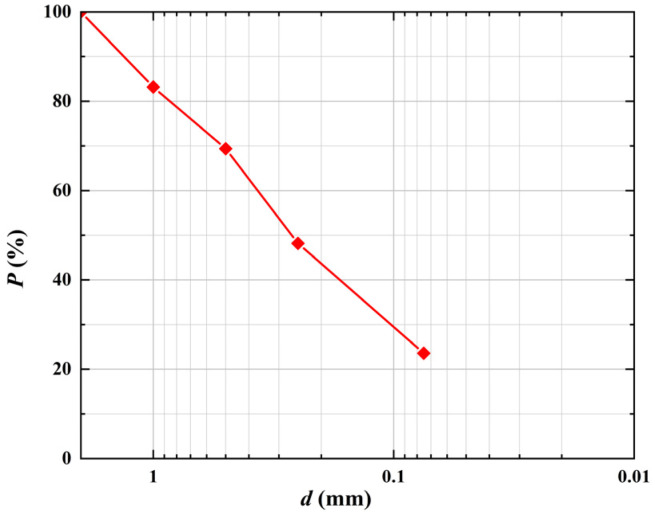
Particle gradation of the soil.

A building site in Huainan City provided the 5–10 m deep clay soil for the test, which has a plastic limit of 24.6% and a liquid limit of 44.8%. It can be categorized as CL with USCS, and its particle size distribution is shown in Figure 1, where *d* indicates the diameter of soil particles and *P* indicates the percentage of the mass of soil less than a certain particle size. The fibers used in the test are PVA fiber and basalt fiber, and the basic parameters are shown in Table 1.

### 2.2. Specimen Preparation and Experimental Methods

**Figure 2:**
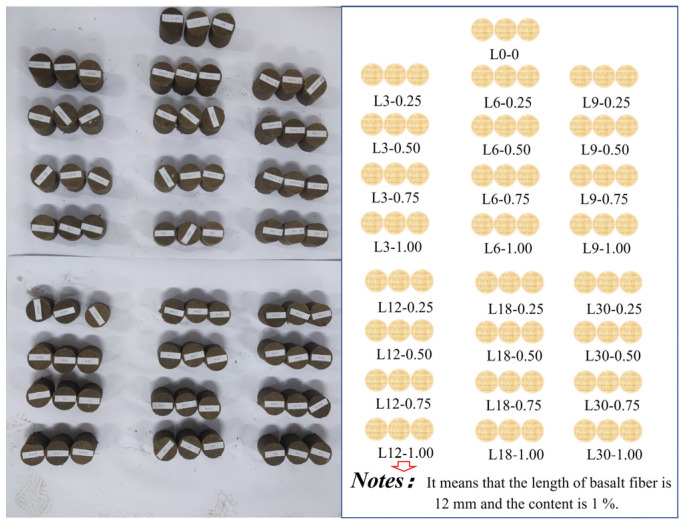
Photos showing the specimens.

The specimens were prepared according to the “Standard for geotechnical method” (GB/T 50123-2019). According to the cement content of 15%, the water cement ratio is 0.5, the PVA fiber (length 12 mm) content is 0.5%, the length of the basalt fiber is 3 mm, 6 mm, 9 mm, 12 mm, 18 mm, and 30 mm, and the basalt fiber content is 0, 0.25%, 0.5%, 0.75%, and 1% (mass fraction). After stirring evenly, a cylindrical specimen with a length of 50 mm and a height of 50 mm was made [36]. We made a total of 25 groups with 3 test blocks in each group for a total of 75 test blocks, as shown in Figure 2. All of the specimens were categorized and numbered with Lx-y, where L represents the length of the basalt fiber added, x represents the length of x mm, and y represents the doping amount of y%. After numbering, the specimens were placed in a standard curing room for 28 days.

**Figure 3:**
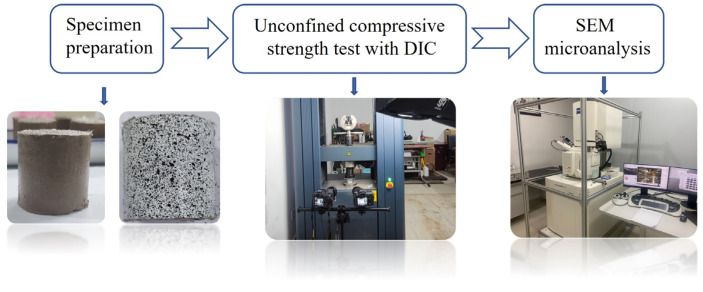
Experimental process.

Before the test, artificial scattered spots were pre-made on the cured cement soil specimens. First, the surface of the specimens was evenly sprayed with white paint. After the white paint was completely dried, the black scattered spots were sprayed on the white bottom with black paint. After the scattered spots were completed, the specimens were placed in a universal testing machine for the UCS test and DIC test. Finally, the compressed specimens were sampled for the SEM test. The whole test process is shown in Figure 3.

#### 2.2.1. Unconfined Compressive Strength (UCS) Test

The UCS test conforms to ASTM D2166/D2166M-16. The loading apparatus is an electronic universal testing machine that is microprocessor-controlled and made by Jinan Zhongluchang Testing Machine Manufacturing Co., Ltd. (Jinan, China) The machine uses a displacement closed-loop control and a loading rate of 1 mm/min. The cured specimens were loaded into the press for UCS testing.

#### 2.2.2. DIC Characterization

**Figure 4:**
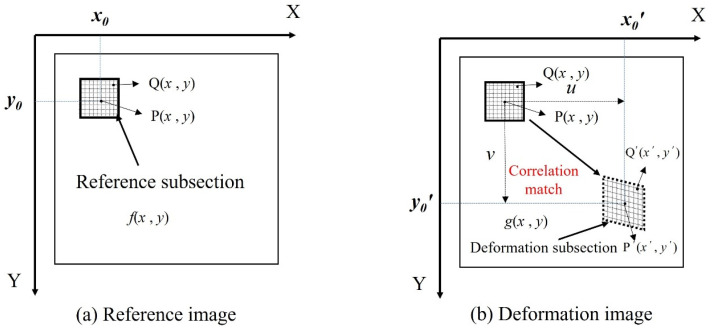
The basic principle of the digital image correlation method.

The creation and evolution of fractures in the specimens were tracked using DIC technology during the UCS test, and the displacement field and strain field on the specimen surface were examined. The basic principle of DIC is shown in Figure 4, assuming that the grey distribution functions for the reference image and the current image are *f* (*x*, *y*) and *g* (*x*, *y*), respectively [37,38], and a square area is selected as the reference subregion within the analysis area of the reference image [39]. Let the subset centroid be P (*x*, *y*), then under the action of the press load, the reference subregion moves to the location of the target subregion and undergoes deformation, while point P moves to point P′, and the trajectory of point P is continuously tracked with the correlation matching function C_LS_ [40]. As shown in Equation (1), the difference between the pixel coordinates of point P before and after deformation is obtained, and the displacement information for point P is obtained.

(1) $$C_{LS}=\sum_{(i,j)\in S}\left[\frac{f(x_{r},y_{r})-f_{m}}{\sqrt{{\sum_{(i,j)\in S}\left(\left(x_{r},y_{r}\right)-f_{m}\right)}^{2}}}-\frac{g(x_{c},y_{c})-g_{m}}{\sqrt{{\sum_{(i,j)\in S}\left(\left(x_{c},y_{c}\right)-g_{m}\right)}^{2}}}\right]$$

The displacement information for other points (T) on the reference subregion can be calculated using Equation (2), where *u_rc_* and *v_rc_* are the displacement results with whole-pixel accuracy obtained with the initial guess [41], and *x* and *y* are the differences in pixel coordinates between points P and Q.

(2) $$\left\{\begin{array}{l} x_{c}=x_{r}+u_{rc}+\left[1+\frac{\partial u(x,y)}{\partial x}\right]\Delta x+\frac{\partial u(x,y)}{\partial y}\Delta y\\ y_{c}=x_{r}+v_{rc}+\left[1+\frac{\partial u(x,y)}{\partial y}\Delta y\right]+\frac{\partial v(x,y)}{\partial x}\Delta x \end{array}\right.$$

#### 2.2.3. SEM Microscopic Testing

After the test, representative specimens were selected for microstructure analysis, and a detailed characterization of basalt and PVA mixed fiber cement soil was performed using field emission scanning electron microscopy (SEM). The SEM test conforms to the ASTM Standards in the SEM. Before the SEM test, a specimen with a maximum radius of less than 10 mm was cut and then put into an oven at 40 degrees Celsius to dry thoroughly. After drying, the gold plating operation was carried out to make it conductive and avoid the charging effect and affecting the imaging quality. Finally, the SEM analysis was carried out using the Zeiss GminiSEM 360 (Jena, Germany).

## 3. Result and Discussion

### 3.1. Effect of Basalt Fiber Length and Content on UCS

**Figure 5:**
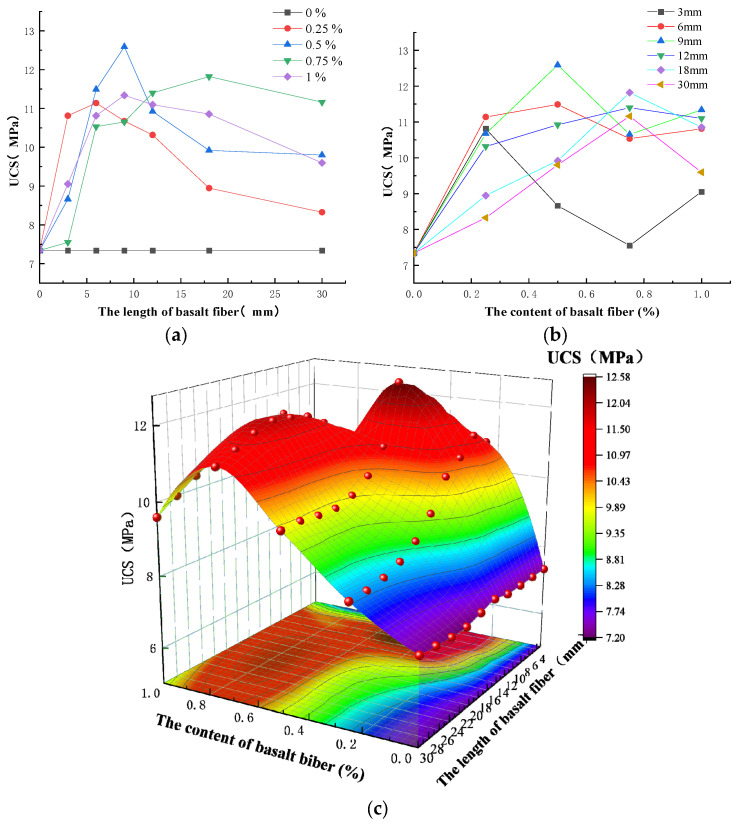
Relationship among UCS and the content and the length of basalt fiber. (**a**) The relationship between UCS and fiber length. (**b**) The relationship between UCS and fiber content. (**c**) The surface diagram for fiber content, fiber length, and UCS.

The association between basalt fiber length and the cement soil specimen UCS is shown in Figure 5a. It can be seen from the diagram that the curve for adding basalt fiber is generally upward. When the content of basalt fiber is constant, the UCS of the cement soil specimens increases first and then decreases with the increase in the length of the fiber. The peak strength in the figure is 12.59 MPa, and the UCS of cement soil without basalt fiber is 7.34 MPa, which is 71% higher than the reference strength.

The association between basalt fiber content and the UCS of cement soil specimens is depicted in Figure 5b. The figure demonstrates that the curvature is often wavy. When the length of the basalt fiber is less than 18 mm, the UCS of the specimens increases, then decreases, and then increases with the increase in the content of the fiber. The UCS of the cement soil specimens initially rises when the basalt fiber length is greater than or equal to 18 mm and then falls when the basalt fiber concentration rises.

According to Figure 5c and the experimental data, it is clear that the basalt fiber length and content both influence the UCS of the hybrid fiber cement soil specimens, and the two factors work in concert to increase the strength of cement soil specimens. The multiple linear regression analysis using the data set shows that the relationship between the UCS and the influencing factors is as follows:

(3) $$\begin{array}{l} \sigma_{UCS}=4.50+27.50c+1.30l-80.1c^{2}+0.14cl-\text{0.17l}{}^{2}+62.74c^{2}+3.05c^{2}l-0.06cl^{2}\\ +0.01l^{4}-11.02c^{4}-2.24c^{3}l-0.01c^{2}l^{2} \end{array}$$

$$R^{2}=0.9317$$

where *σ_UCS_* is the unconfined compressive strength, MPa; *c* is the content of basalt fiber, %; and *l* is the length of basalt fiber, mm.

Both basalt and PVA fibers have greater tensile strength [42]; therefore, in the UCS test, the fiber is pulled out during the failure of the cement soil specimen, so the UCS of the cement soil specimen largely depends on the magnitude of the bonding force between the cement soil and the fiber. In general, with the increase in fiber content, there is an interlocking effect, an anchoring effect, and a friction effect. The interlocking effect is mainly reflected in the unevenness of the cement soil specimen, and the random distribution of the fiber will be embedded with the soil, thus preventing the development of soil deformation during the process of soil failure. During the anchoring effect, the fiber randomly passes through the soil, which can provide tension between the soil particles inside the specimen, and can form a compression zone within a certain range around the fiber. At the same time, the fiber and the fiber will form a compression band. The compression band and the compression band are closely overlapped so that the integrity of the specimen is enhanced, and the bearing capacity is improved. The friction effect mainly depends on the friction between the fiber and the soil particles [43], and the friction coefficient of the interface is the key to whether the friction effect can play a role.

**Figure 6:**
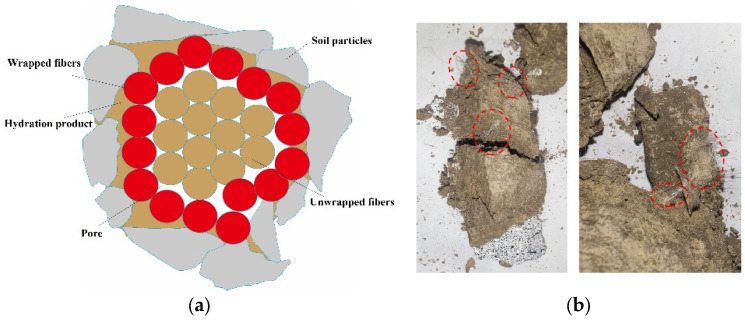
The fiber bundle phenomenon. (**a**) State diagram showing fibers in cement soil. (**b**) Fiber bundles in cement soil.

The degree of fiber dispersion also significantly affects the UCS of cement soil. When the fibers are dispersed more evenly in the cement soil, the fiber in a monofilament state is similar to the steel bar in building materials, which plays a role in micro-reinforcement when micro-cracks appear, which can improve the strength and stability of the material [42,44]. However, at higher basalt fiber content levels, the fiber bundle phenomenon will appear inside the specimen. As shown in Figure 6, the fiber cannot be fully dispersed and opened to become a monofilament state. The higher the content of basalt fiber, the more serious the fiber bundle phenomenon, and the smaller the bonding area between the fiber and the cementitious material in the cement soil. In addition, the fiber bundle may cause a weak surface on the hole and cause stress concentration, thus reducing the strength of the cement soil material.

According to Figure 5, under the existing test conditions, the UCS of the cement soil specimens fluctuates with the change in the basalt fiber content and length. It is clear that neither the basalt content nor basalt fiber length necessarily correlate with higher performance. When the content of basalt fiber is 0.5% and the length is 9 mm, the maximum UCS is 12.59 MPa, and the UCS of cement soil with basalt fiber is higher than it is without basalt fiber.

### 3.2. The Influence of Basalt Fiber Length and Content on the Stress–Strain Curve

**Figure 7:**
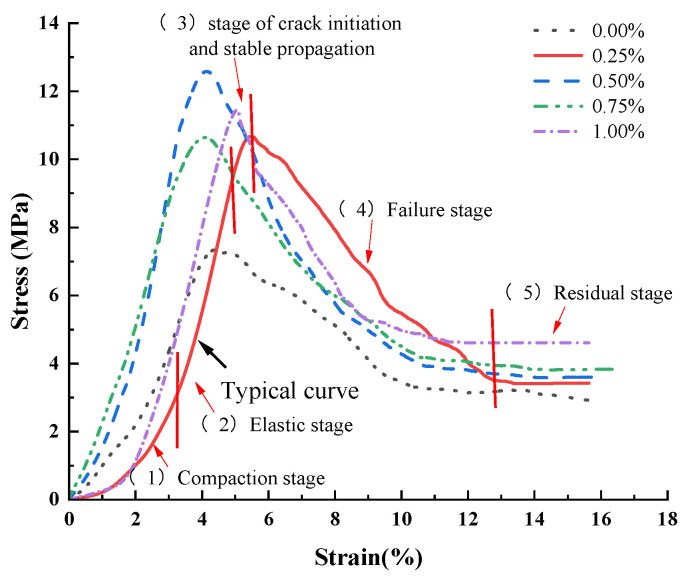
Stress–strain curves for cement soils with different basalt fiber content (9 mm).

Figure 7 shows that the overall change trend in the stress–strain curve for the specimen is roughly the same under the condition of different contents and different lengths of basalt fiber, and the stress–strain curve for the cement soil in the compression process can be similarly divided into five stages [43,45,46,47]. (1) During the compaction stage, the cement soil specimen is subjected to an external load, and the fine particles are rearranged. The pores inside the specimen are gradually compacted, and the stress–strain curve is relatively slow. (2) During the elastic stage, when the cement soil specimen is compacted, the stress–strain curve for this stage is approximately linear, and the internal structure of the cement soil specimen is stable. (3) During the stage of crack initiation and stable propagation, when the stress in the cement soil specimen reaches the crack initiation stress, the crack develops steadily with the increase in stress. (4) During the failure stage, with the further increase in stress, the crack develops faster and faster after the stress reaches the damage stress. When the peak stress is reached, a penetrating crack is formed inside the specimen. When the test force exceeds the maximum stress that the specimen can withstand, damage occurs, resulting in a sharp decrease in the stress–strain curve. (5) During the residual stage, the stress–strain curve for the specimen decreases slowly, which can be approximated as a horizontal line.

In order to make the text clearer, the secondary factors were discarded from the analysis, and the basalt fiber content (0.5%) and length (9 mm) with the maximum UCS in this experiment were selected for analysis. It can be seen from Figure 7 that when the length of the added fiber is 9 mm, and the content of added fibers is 0%, 0.25%, 0.5%, 0.75%, and 1%, the peak stress and residual stress in the cement soil specimens are 7.33 MPa and 2.92 MPa; 10.67 MPa and 3.42 MPa; 12.59 MPa and 3.60 MPa; 10.65 MPa and 3.83 MPa; and 11.45 MPa and 4.61 MPa, respectively.

With the increase in the fiber content, the UCS of the cement soil specimens increases first, then decreases, and then increases with the increase in fiber content, and the residual stress increases continuously. Compared with the specimens without basalt fiber, the UCS and residual strength of the cement soil specimens with basalt fiber are improved to varying degrees, showing stronger plasticity. This is because the basalt fiber dispersed in cement soil can form a network structure, which can be combined with soil particles and hydration products. When cracks occur, the force between the hydration products and cementitious materials fails, and the existence of the fiber produces new bridging forces [35,48], thus preventing the expansion of cracks and greatly reducing the possibility of brittle damage to the specimens. This greatly reduces the possibility of brittle failure of the soil specimen and improves the reliability of the cement material. Nevertheless, when the fiber content exceeds the critical level (0.5% in this test), there is a resulting reduction in the fiber bridging effect and, hence, a reduction in the peak strain.

**Figure 8:**
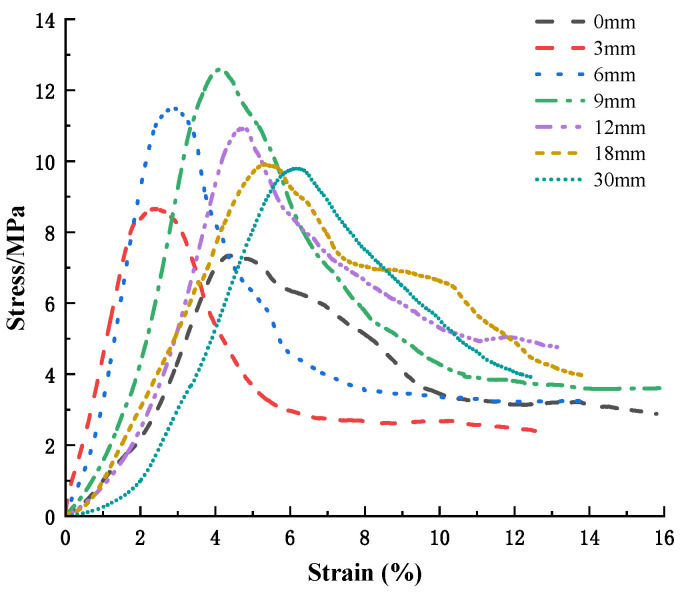
Stress–strain curves for cement soils at different fiber lengths with a basalt fiber content of 0.5%.

It can be seen from Figure 8 that when the basalt content is 0.5% and the fiber length is 3 mm, 6 mm, 9 mm, 12 mm, 18 mm, and 30 mm, the peak stress and strain are 8.66 MPa and 0.02447; 11.49 MPa and 0.02924; 12.59 MPa and 0.04146; 10.92 MPa and 0.04757; 9.92 MPa and 0.05328; and 9.8 MPa and 0.06174, respectively. As the length of the fiber increases, the peak tension in the cement soil initially rises and then falls. After adding basalt fiber, the failure strain increases with the increase in fiber length, showing stronger toughness behavior.

### 3.3. The Effect of Basalt Fiber Length and Content on the Cracking of Cement Soil Specimens

**Figure 9:**
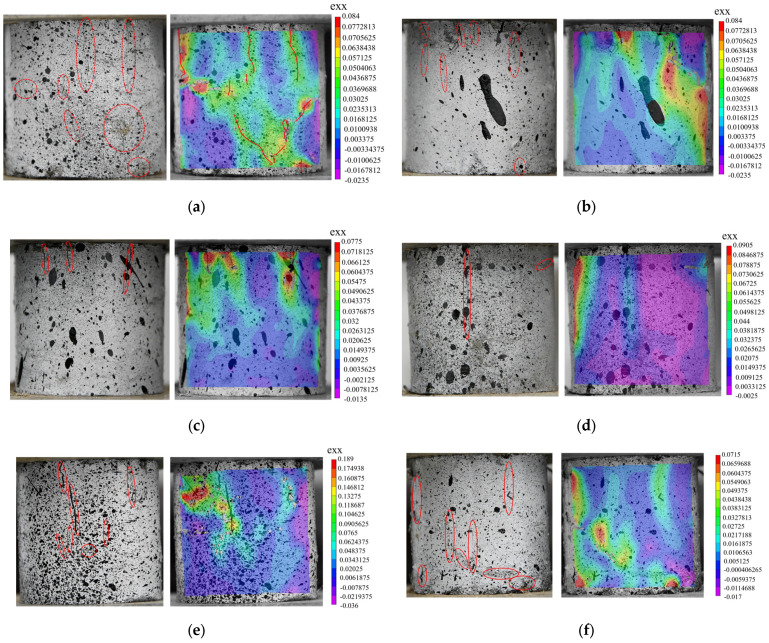

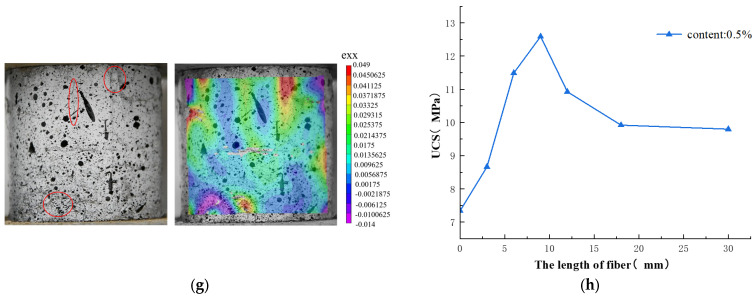
The horizontal strain field and stress at the peak stress when the basalt fiber content is 0.5%. (**a**) L0-0.5 (without basalt fiber); (**b**) L3-0.5; (**c**) L6-0.5; (**d**) L9-0.5; (**e**) L12-0.5; (**f**) L18-0.5; and (**g**) L30-0.5. (**h**) The relationship between UCS and fiber length when the basalt fiber content is 0.5%.

DIC technology is a simple and universal method for analyzing full-field strain. In order to make the text clearer, the secondary factors were discarded from the analysis. The basalt fiber content (0.5%) and length (9 mm) with the maximum UCS in this experiment were selected for analysis. The horizontal strain cloud diagram at the peak stress of the cement soil specimen under the two conditions was analyzed, and the true failure state and strain cloud diagram for the specimen are compared. As shown in Figure 9, the left side shows the true failure state of the specimen, the red line marks the surface crack in the specimen, and the right side shows the horizontal strain field cloud diagram. The cloud diagram for the horizontal strain field not only shows the location of the crack but also characterizes the relative size of the local crack with the color, which can be regarded as the crack distribution diagram to a certain extent. As can be seen in Figure 9:

(1) Compared with the cement soil specimen without basalt fiber, after the addition of basalt fiber, the number and length of cracks on the surface of the cement soil specimen are reduced to varying degrees after reaching the peak stress. This indicates that the addition of basalt fiber more or less increases the crack resistance of the cement soil specimen and improves the strength of the cement soil specimen [47], which is consistent with the results from the UCS test.
(2) In the horizontal strain cloud diagram, all the places where strain concentrations appear have developed cracks or are about to develop cracks in the following compression process. Comparing the UCS in Figure 9e, it is found that the higher the UCS, the less the strain concentration in the horizontal strain cloud diagram.
(3) In the UCS test, the fractures created during the failure process of the cement soil specimens are primarily vertical, and the higher the strength, the fewer cracks. Nevertheless, the length and breadth of the cracks are frequently comparatively larger and wider. This is because when there are fewer cracks, the energy in the compression process is dissipated from fewer cracks, resulting in greater damage in a single crack.
(4) The deformation properties of fiber-reinforced composites are closely related to the number and width of cracks. When the crack width is not much different, more cracks will lead to greater deformation. According to the crack situation in Figure 9, the compressive strain of the cement soil specimen decreases initially and subsequently increases with the increase in fiber length.

**Figure 10:**
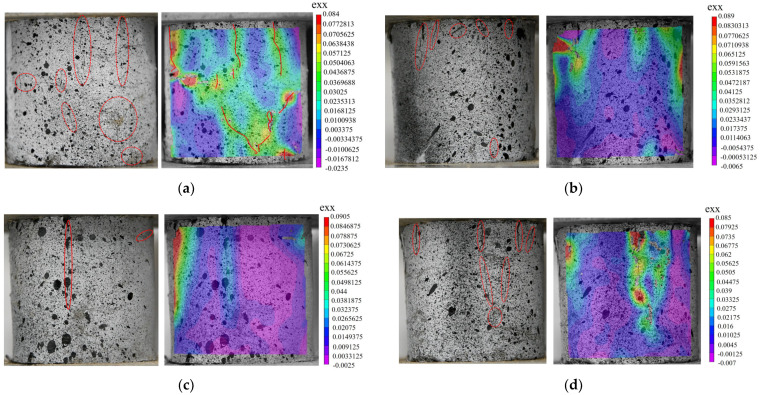

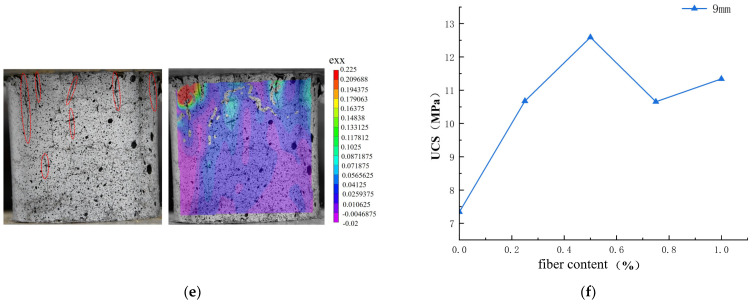
The horizontal strain field and stress at the peak stress when the length of the basalt fiber is 9 mm. (**a**) L9-0 (without basalt fiber); (**b**) L9-0.25; (**c**) L9-0.5; (**d**) L9-0.75; and (**e**) L9-1. (**f**) The relationship between UCS and fiber content when the basalt fiber is 9 mm.

As can be seen from Figure 10:

(1) When the length of the basalt fiber is 9 mm, the cracking degree of the cement soil specimen at the peak stress decreases first, then increases, and then decreases with the increase in fiber content, which is contrary to the UCS of cement soil shown in Figure 10f. During the UCS test, when the cement soil specimen reaches the peak stress, there are more cracks on the surface, and the weaker the strength of the specimen. We can see the number and degree of strain concentration using DIC characterization analysis and use it to judge the failure strength of the fiber cement soil specimen.
(2) After the addition of basalt fiber, there will be more micro-cracks at the top of the cement soil specimen when the peak stress is reached. As shown in Figure 10b–f, the number of micro-cracks at the top of the specimen is negatively correlated with the strength of the specimen, the UCS of the specimen is maximum at 0.5% (9 mm) of basalt fiber admixture, and the top of the specimen has good integrity.

In summary, DIC technology can not only clearly identify the micro-cracks that are difficult to see with the naked eye using the strain cloud diagram and the location of stress concentration, but it can also evaluate the degree of local deformation in the material. The results of the cloud diagram are consistent with the results for the UCS. Therefore, DIC technology is a feasible method for monitoring the compressive failure of cement–soil materials. It can be a powerful tool to provide early warning before the formation of destructive cracks in a structure [49].

### 3.4. SEM Microscopic and Cement Soil Curing Mechanism Analysis

**Figure 11:**
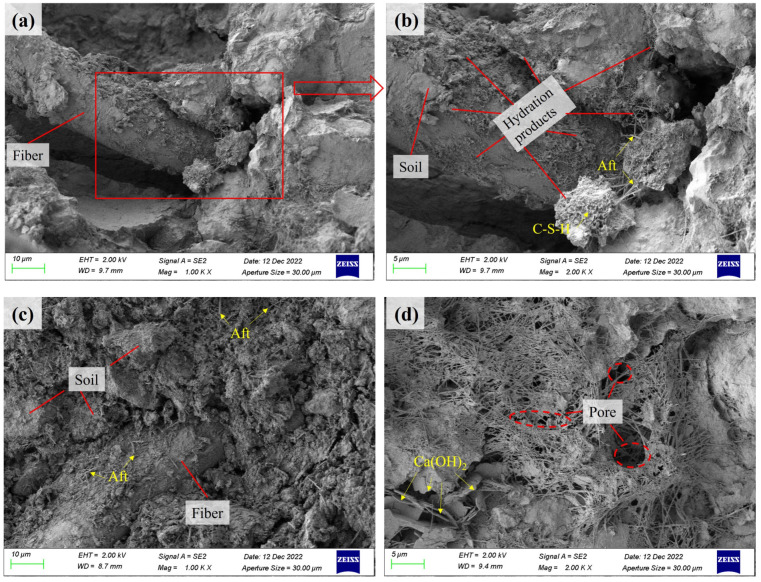
SEM image showing cement soil (0.5% and 9 mm basalt fiber). (**a**) 1000×; (**b**) 2000×; (**c**) 1000×; and (**d**) 2000×.

To understand the reinforcing effect of basalt fiber on PVA fiber cement soil further, we observed its microstructure using an SEM test. The fiber is mainly in three states in the cement soil matrix: a completely wrapped state, a partially debonded state, and a completely pulled-out state. When the fiber is in a completely wrapped state, as shown in Figure 11d, with a further increase in the load, the cracks begin to propagate along the weak interface between the particles and the fibers in the specimen, while the load on the particles in the soil is transferred to the fibers. With a further increase in the external load, the cracks between the soil particles and the hydration products of the cement are further extended, the cementation among them fails and the forces inside the specimen are gradually transferred to the fibers, and the fibers are gradually pulled out with the expansion of the cracks and the increase of the load, as shown in Figure 11a.

Figure 11a shows a micrograph of the cement soil with a basalt fiber content of 0.5% at 1000 times. It can be seen that there are a large number of hydration products of cement around the fiber, such as C-S-H, Aft, etc. The fibers and soil particles are joined together by the hydration products of cement, while the internal voids in the cement soil are filled with the hydration products. This reduces the porosity of the specimen, increases the contact area between the fiber and the cement soil, and then increases the cohesion and friction between the fiber and the particles and the resistance to external loads [50].

According to the results of the UCS, the data from DIC analysis, and the results of scanning electron microscopy (SEM), it can be concluded that the curing mechanism for basalt and PVA hybrid fiber cement soil mainly depends on the role of cement. The role of cement is mainly manifested in the hydration products such as C-S-H and Aft generated by the hydration reaction, pozzolanic reaction, and ion exchange reaction. The main chemical composition of ordinary Portland cement is CaO, SiO_2_, Al_2_O_3_, and Fe_2_O_3_, and the main mineral composition is 2CaO • SiO_2_, 3CaO • SiO_2_, 3CaO • Al_2_O_3_ and 4CaO • Al_2_O_3_ • Fe_2_O_3_. The hydration reaction that occurs in the specimens during the curing process is mainly a chemical reaction between the four mineral components in the cement with water to form C-S-H, C-A-H, Aft(3CaO • Al_2_O_3_ • 3CaSO_4_ • 31H_2_O), and Ca(OH)_2_, as shown in Equations (4)–(7).

It can be seen from Figure 11b–d that a large number of fine abrasive fibrous hydration products (C-S-H) agglomerate together to form a honeycomb structure or a network structure. The needle-like Aft crystals are closely wrapped with cement and soil particles. These hydration products effectively restrict the relative movement between fibers and cement aggregates in the cement soil specimens, thus greatly increasing the strength of the specimen.

**Figure 12:**
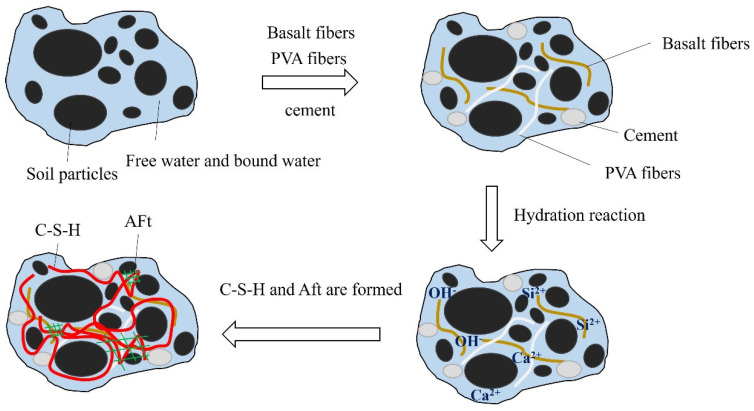
Mechanism diagram showing cement curing for cement soil materials.

During the hydration reaction, there will be an exothermic phenomenon. The increase in temperature will cause a small amount of water in the soil to evaporate, causing a relative increase in the volume of soil particles and other solids. At the same time, the generated Ca(OH)_2_ can effectively fill the pores. As a result, the strength of the soil is improved, and the compressibility is reduced. Due to the occurrence of the hydration reaction, OH^−^ ions precipitate, the concentration of OH^−^ increases, and Si^2+^ ions in silt soil react with Ca^2+^ and OH^−^ ions to form hydrated calcium silicate gel (C-S-H), which is an ionic reaction, as shown in Equation (8). Active SiO_2_ reacts with Ca(OH)_2_ to form C-S-H, which is a pozzolanic reaction, as shown in Equation (9). The microscopic evolution mechanism for cement-to-cement soil curing is shown in Figure 12.

(4) $$2\left(2\mathrm{CaO}•{\mathrm{SiO}}_{2}\right)+4H_{2}O\;\to \;3\mathrm{CaO}•2{\mathrm{SiO}}_{2}•3H_{2}O+\mathrm{Ca}{\left(\mathrm{OH}\right)}_{2}$$

(5) $$2\left(3\mathrm{CaO}•{\mathrm{SiO}}_{2}\right)+6H_{2}O\;\to \;3\mathrm{CaO}•2{\mathrm{SiO}}_{2}•3H_{2}O+3\mathrm{Ca}{\left(\mathrm{OH}\right)}_{2}$$

(6) $$2\left(3\mathrm{CaO}•{\mathrm{Al}}_{2}O_{3}\right)+20H_{2}O\;\to \;4\mathrm{CaO}•{\mathrm{Al}}_{2}O_{3}•12H_{2}O+2\mathrm{Cao}•{\mathrm{Al}}_{2}O_{3}•8H_{2}O$$

(7) $$3\mathrm{CaO}•{\mathrm{Al}}_{2}O_{3}•6H_{2}O+3({\mathrm{CaSO}}_{4}•2H_{2}O)+19H_{2}O\;\to \;3\mathrm{CaO}•{\mathrm{Al}}_{2}O_{3}•{3\mathrm{CaSO}}_{4}•31H_{2}O$$

(8) $${\mathrm{SiO}}_{2}+\mathrm{Ca}{\left(\mathrm{OH}\right)}_{2}+H_{2}O\;\to \;\mathrm{CaO}•{\mathrm{SiO}}_{2}•H_{2}O$$

(9) $${\mathrm{SiO}}_{2}+2{\mathrm{OH}}^{-}\to H_{2}{\mathrm{SiO}}_{4}^{2-}$$

## 4. Conclusions

In this paper, the UCS test was carried out by adding basalt fiber at different contents and different lengths to cement soil, and the results of the experiment were further analyzed using the DIC test and SEM test. The law of fiber content and length on the UCS change in cement soil was obtained. The main conclusions are as follows:

(1) The PVA fiber cement soil specimen with basalt fiber (0.5%, 9 mm) has the largest increase in UCS, which is 71% higher than the UCS of cement soil specimens without basalt fiber. Other lengths (3 mm, 6 mm, 9 mm, 12 mm, 18 mm, 30 mm) and contents (0%, 0.25%, 0.5%, 0.75%, 1%) of basalt fiber more or less increased the UCS of the cement soil specimen. Multiple linear regressions were performed on the UCS, fiber length, and fiber content. The relationship between the three was obtained.
(2) The stress–strain curve for fiber cement soil during the compression process can be divided into five stages: the compaction stage, the elastic stage, the stage of crack initiation and stable propagation, the failure stage, and the residual stage. When the fiber length is 9 mm, the residual stress of the cement soil specimens increases with the increase in fiber content. When the basalt fiber content is 0.5%, the failure strain increases with the increase in fiber length, showing stronger toughness.
(3) With the addition of basalt fiber, the number and size of cracks on the surface of the cement soil specimen are reduced to varying degrees when the peak stress is reached. Combined with the strain cloud diagram obtained using DIC technology, the location of cracks can be found simply and efficiently. According to the location of the strain concentration in the cloud diagram, the cracks that are difficult to see with the naked eye can be found, and the location of the cracks that will be generated can be predicted.
(4) In the SEM diagram, the dense network structure can be seen inside the specimen, and the periphery of the fibers is closely wrapped with the hydration products of the cement. The hydration products of the cement fill in the pores to enhance the compactness and strength of the specimen. The overall network structure of the space is obviously formed, which makes the soil skeleton have better integrity and cementation and enhances the strength of the specimen.

In summary, the addition of PVA fiber can largely improve the strength and crack resistance of cement soil material, and the optimum doping amount (0.5% and 12 mm basalt fiber and 0.5% and 9 mm PVA fiber) obtained from this experiment can provide a reference for the future application of cement soil material in engineering. In addition, the study of cement materials is not only limited to static mechanical properties but the dynamic mechanical properties can also be analyzed using the split Hopkinson pressure bar test. DIC technology is a simple and efficient way to analyze the strain field and displacement changes on the surface of an object. It should be widely promoted in the field of material properties testing and can be applied in geotechnical engineering. It can be extended to the whole geotechnical engineering field, coal and rock mining field, automotive industry field, and biomedical field.

## Figures and Tables

**Table 1:** Mechanical property parameters of the fibers.

Ingredients	Types	Length/mm	Diameter/μm	Tensile Strength/MPa	Tensile Elasticity Modulus/GPa	Fracture Elongation/%
PVA fiber	Monofilament	12	15.1	≥1500	≥35	6.9
Basalt fiber	Monofilament	3, 6, 9, 12, 18, 30	18.5	≥2000	≥85	2.92

## Data Availability

The data presented in this study are available on request from the corresponding author.
